# Developing Galectin-3-Targeted Therapeutics in Diseases: Three Distinct Galectin-3 Segments for Targeting

**DOI:** 10.1017/erm.2026.10060

**Published:** 2026-06-15

**Authors:** Paulina Sindrewicz-Goral, Oluwatobi Adegbite, Jessie Y. Yu, Lu-Gang Yu

**Affiliations:** 1 Department of Biochemistry, Cell and Systems Biology, University of Liverpool, UK; 2 Aintree University Hospitals NHS Foundation Trust, Liverpool, UK

**Keywords:** cancer, drug development, fibrosis, galectin-3, inflammation, small molecule inhibitor

## Abstract

The *β*-galactoside-binding protein galectin-3 is currently a hotly pursued therapeutic target in cancer, inflammation and fibrosis-associated diseases due to its multi-mode actions and broad impact on the pathogenesis and progress of the diseases. Various natures of galectin-3 inhibitors have been developed and investigated, and several have shown promising results in early-phase clinical trials. All these galectin-3 antagonists were designed to target the canonical carbohydrate-binding site, the S-face, of the galectin-3 carbohydrate recognition domain (CRD). This review discussed the current galectin-3 antagonists and explored their modes of actions, focusing particularly on their targeting regions on galectin-3. It discussed the tri-modular structure of galectin-3 and the roles of different segments in galectin-3 actions. It proposed that, in addition to the canonical carbohydrate-binding sites on the S-face, the non-canonical carbohydrate-binding interface, the F-face of the galectin-3 CRD as well as its flexible N-terminal domain are also targetable in the design of galectin-3-targeted therapeutics. Given the high degree of structural similarities of CRDs among galectin family members but unique nature of galectin-3 N-terminus, antagonists developed against the N-terminal domain of galectin-3 can potentially offer greater target specificity by avoiding cross-reactivity with other galectin members. Antagonists that can interact with more than one segment of galectin-3, or a combination of antagonists against different galectin-3 segments, may potentially provide improved efficacy and therapeutic effectiveness for treatment of galectin-3-mediated pathologies and diseases.

## Introduction

Galectins are a family of 15 carbohydrate-binding mammalian lectins, each of which contains one or two highly conserved carbohydrate- recognition domains (CRDs) that bind to galactose-terminated carbohydrates (Ref. 1). Galectins are divided into three subgroups based on their structural architecture: the proto-, chimera- and tandem-repeat types (Hirabayashi and Kasai 1993). The prototype galectins in humans include galectin-1, -2, -7, -10, -13, -14 and -16. Proto-type galectins each contain one CRD but they all can form non-covalent, predominantly symmetric, homodimers with the exception of galectin-13 which dimerizes via disulphide bonds (Refs 85; Su et al. 2018). The tandem-repeat type galectins in human include galectin-4, -8, -9 and -12, each containing two CRDs within a single peptide chain that are connected by a short linker region. Galectin-3 is the sole chimera-type galectin and has a CRD at its C-terminal and a highly flexible N-terminal sequence which are connected by a collage-like repeat region. Despite their remarkable sequence and structural resemblances, the carbohydrate-binding preferences for complex glycoconjugates among galectin members vary considerably (Ref. 2). These differences among galectins in carbohydrate-binding specificities/recognition allow each galectin to be able to interact with a discrete spectrum of glycans, which can lead to different biological effects in pathophysiological conditions (Ref. 3). As a result of their pleotropic actions via binding to a divergent glycans and receptors, changes of galectin expression affect divergent biological activities in various pathological processes (Refs 4, 5, 6).

As the exclusive chimera-type galectin, galectin-3 is expressed by various cell types including immune cells, epithelial cells and fibroblasts (Refs 7, 8). Galectin-3 can bind to various galactose-terminated glycans extracellularly and also to non-glycosylated proteins intracellularly through its carbohydrate-recognition domain (CRD) (Refs 8, 9). This ability allows galectin-3 to act as a multi-functional molecule to regulate divergent biological activities in cell–cell and cell–environment communications.

Overexpression of galectin-3 is commonly observed in a number of pathological conditions, including cancer (Refs 7, 10), chronic and acute inflammation (Ref. 11) and tissue fibrosis (Refs 12, 13, 14, 15, 16, 17, 18). Change of galectin-3 expression is often associated with disease progression and poorer prognosis (Refs 19, 20, 21). Over the past decade, a huge body of evidence has shown that galectin-3 is an important promoter in cancer development, progression and metastasis and an active regulator in prolonged inflammation and formation of organ/tissue fibrosis.

The profound impact of galectin-3 in promoting the pathogenesis and progression of such a broad range of diseases has prompted substantial efforts over the past decade to develop galectin-3-targeted therapeutics in those disease areas (Refs 4, 22, 23). A number of galectin-3 inhibitors and antagonists have been identified and showed effects on blocking galectin-3-mediated actions in vitro and in animals (Ref. 4) and several have demonstrated encouraging results in phase I/II clinical trials in cancer and fibrosis-associated diseases (Ref. 22).

### Galectin-3 tri-modular structure

**Figure 1.**
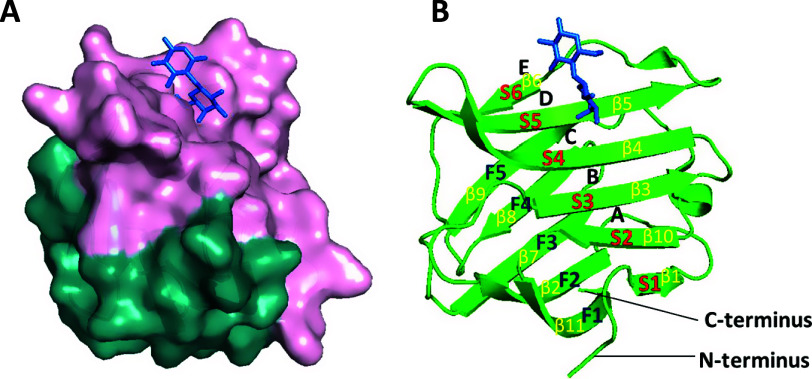
Schematic representation of galectin-3 CRD. *Note*: **A.** The CRD of galectin-3 (3ZSJ) is shown in cartoon representation with lactose molecule (blue) in stick representations. The concave S-face with the carbohydrate-binding site is coloured in light pink, whereas the convex F-face is shown in teal. **B.** Carbohydrate-binding subsites A–E as well as S-face S1–S6 (β1, β10, β3, β4, β5 and β6) strands and F-face F1–F6 (β11, β2, β7, β8 and β9) strands are indicated. Lactose molecule predominantly occupies the C and D subsites. Figure 1. long description.

Galectin-3 bears a distinct tri-modular architecture that is different from the other galectin members. Galectin-3 possesses a lengthy and flexible N-terminal domain (approximately 112 amino acids in total) that is rich in glycine, proline and tyrosine residues and is fused to a C-terminal carbohydrate-recognition domain (CRD, 138 amino acids) through a linker region. The galectin-3 CRD is arranged into a globular β-sandwich structure formed by two antiparallel β-sheets made up of 11 strands (Figure 1). Six strands (S1–S6, made up by strands β1, β10, β3, β4, β5 and β6) form the concave S-sheet/face of the carbohydrate-binding site, whereas another five strands (F1–F5, made up by strands β11, β2, β7, β8 and β9) form the opposing F-sheet/face convex site (Refs 24, 25). The carbohydrate-binding site of galectin-3 is long enough to accommodate a linear tetrasaccharide and can be divided into four main subsites A to D and an additional, less recognized subsite E (Figure 1B) (Ref. 26). Subsite C is the defining β-galactoside-binding site with a conserved sequence motif of seven amino acids, which forms stacking interactions with galactose, whereas subsite D serves as the second part of the galectin-3 canonical disaccharide-binding site. Both subsites form the core of galectin-3 CRD, which is highly conserved throughout the galectin family (Ref. 8). The sequence extension of CRD beyond the C and D regions greatly increases the binding affinity of galectins with carbohydrates, especially with larger glycans. For example, poly-N-acetyl-d-lactosamine neo-glycoconjugates, which can interact with subsites A and B in addition to the core C and D subsites, have a much higher binding affinity with galectins than monovalent N-acetyl-d-lactosamine (LacNAc), which forms interactions with residues only in subsites C and D (Refs 8, 27).

**Figure 2.**
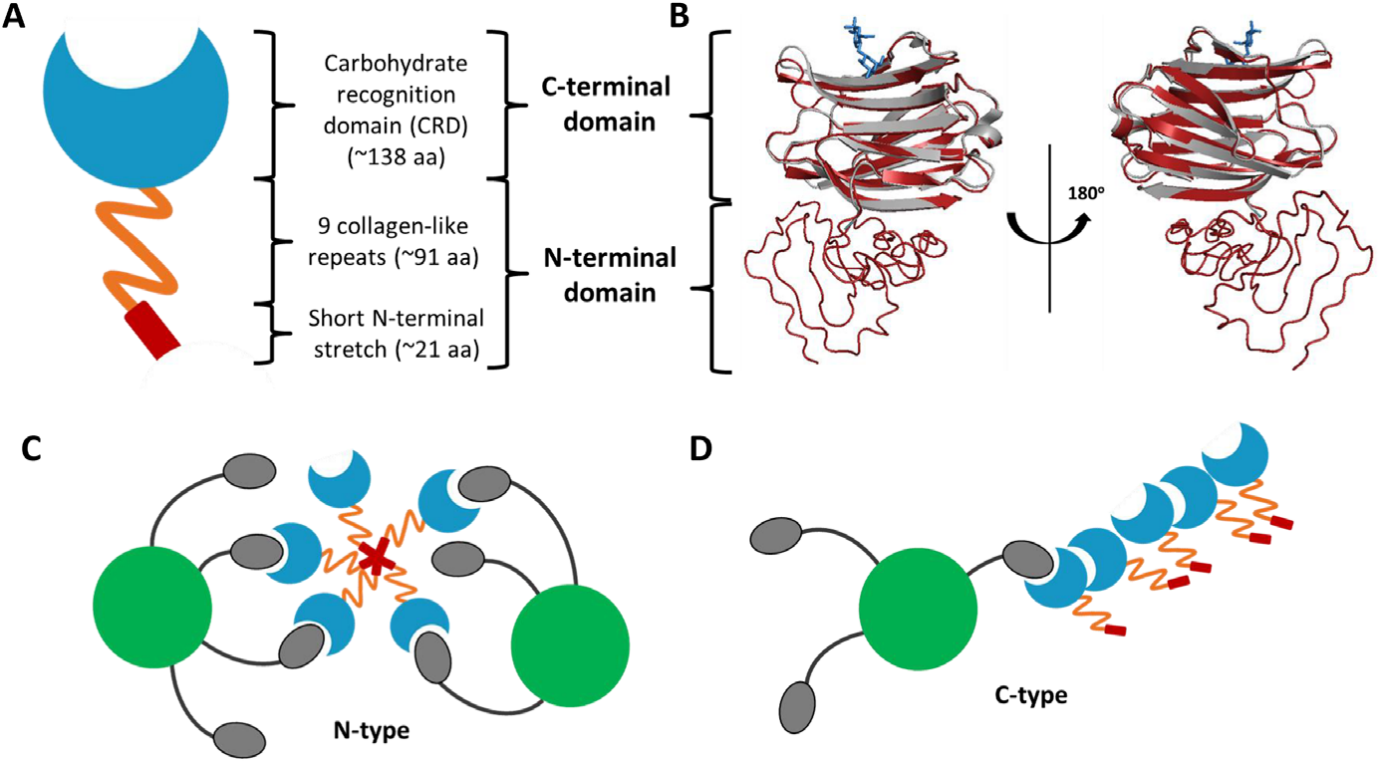
Schematic representation of the monomeric and oligomeric structures of galectin-3 without and with presence of binding ligands. *Note*: **A.** Tri-modular structure of galectin-3 includes very short N-terminal stretch of 21 amino acids with two phosphorylation sites, collagen-like repeat domain and carbohydrate recognition domain. **B.** Predicted by I-TASSER structure of the full-length galectin-3 is shown in cartoon representation (red) and overlaid with the crystal structure of galectin-3 CRD (3ZSJ) (grey). Lactose molecule in blue stick representation is also shown. Models of galectin-3 self-association into dimers and higher oligomeric structures in the presence of binding ligand is shown in **C** and **D**: N-type multimerization mediated by the N-terminal domains (**C**) and C-type self-association via C-terminal domains (**D**). Figure 2. long description.

Although the CRD domain of galectin-3 is well defined, the structure of full-length galectin-3 has not yet been determined because of the technical challenges to determine the long and highly flexible N-terminal domain. The predicted structure of the full-length galectin-3 by I-TASSER is illustrated in Figure 2. The predicted structure of the galectin-3 CRD (shown in red in Figure 2B) matches very well with the structure determined by X-ray crystallography (grey). The N-terminal domain of galectin-3 is composed of an N-terminal segment of approximately 21 amino acids with two serine phosphorylation sites at positions 6 and 12, followed by an elongated, non-triple-helical collagen-like and structurally aperiodic tail of about 90 amino acids (Refs 28, 29). This unique galectin-3 region is made up of nine collagen-like repeats rich in proline, glycine and tyrosine residues (Ref. 29). This conformationally flexible N-terminal domain is responsible for galectin-3 multimerization upon multivalent ligand binding. The first 12 amino acid residues within the N-terminal fragment, so called the leader sequence, are particularly important for the activity of galectin-3 multimerization (Ref. 30). In addition, this short sequence also governs cellular compartmentalization and plays a role in galectin-3 secretion (Ref. 31). Despite lacking carbohydrate-binding activity, the N-terminal domain has been shown to contribute to galectin-3 binding by oligosaccharide (Ref. 32).

The N-terminal domain of galectin-3 can undergo post-translational modification by phosphorylation that contributes to intracellular galectin-3 movement. Galectin-3 export from the nucleus to the cytoplasm is dependent on phosphorylation of Ser6 at the N-terminus (Refs 33, 34). Phosphorylation of Ser6 has been shown to significantly reduce galectin-3 binding to laminin and asialomucin (Ref. 35). Besides serine residues, tyrosine residues at positions 79, 107 and 118 can also be phosphorylated by c-Abl kinase, with Tyr 107 being the main target (Ref. 36). In addition, the N-terminal domain harbours cleavage sites for various collagenases and elastases (Ref. 37) as well as MMP-2 and MMP-9 matrix metalloproteinases (Refs 38, 39). These enzymes cleave galectin-3 at Ala62-Tyr63 position and reduce the self-association abilities of the protein, thereby modulating its biological functions (Ref. 39).

### Intermolecular self-association of galectin-3

There is considerable ambiguity in the literature regarding the existence of multimeric states of galectin-3. Although galectin-3 has been reported to exist in several different oligomeric forms, the actual quaternary structure of galectin-3 remains unclear and has been a subject of dispute for many years. Galectin-3 was shown on many occasions, predominantly by early research using gel filtration chromatographic methods, to exist in solution in a monomeric form (Refs 38, 40, 41, 42). Galectin-3 was reported to remain monomeric mostly at lower concentrations but also in the presence of monovalent ligand such as LacNAc (Ref. 42). Significant efforts have been made to investigate why despite possessing a CRD structure identical to prototypic galectins which form homodimers, galectin-3 does not self-associate into similar dimeric state. A comparison of crystal structures of galectin-3 with galectin-1 and -2 provided an explanation on why this chimeric galectin does not show the characteristic twofold symmetric dimer assembly. Detailed structural analysis uncovered several features displayed by galectin-3 such as reduced apolar nature of the dimer interface and obstruction of the canonical dimerization site by residues at the end of the N-terminal domain like Leu114, Ile115 and Val116, which are absent from the prototypic galectins. These structural differences were made accountable for the inability of galectin-3 monomers to associate into symmetric dimers (Ref. 41). Second possible explanation on why galectin-3 does not dimerize in the same manner as prototypic galectins stems from the presence of the flexible N-terminal domain, which was suggested to interact with the C-terminal domain and thus, possibly obstruct formation of dimers and higher multimers (Refs 32, 43, 44). Despite all this evidence supporting galectin-3’s inability to dimerize, several early reports stated formation of galectin-3 homodimers, many of them however were observed at high protein concentrations (Ref. 45). One earlier study suggested that galectin-3 dimerization is mediated by cysteine residues by forming disulphide bridges (Ref. 46). Alternative dimerization model was suggested for galectin-3 self-association via C-terminal domain of one monomer. However, the binding interface on the second molecule remained unknown. As the truncated form of galectin-3 consisting of only C-terminal domain (Gal-3C) that did not bind immobilized Gal-3C, it was suggested that the second binding site was within the N-terminal domain. What is more, the binding was carbohydrate dependent as presence of lactose blocked these homophilic interactions, indicating that the conventional carbohydrate-binding site was involved in self-association (Ref. 47).

Galectin-3 is well known for its ability to form multimers. N-type self-association, schematically presented in Figure 2C, which occurs upon multivalent ligand binding and contributes to its cross-linking ability, is generally accepted and validated by numerous studies (Refs 48, 49, 50). In 2004, Ahmad et al. reported that galectin-3 precipitates in pentameric state in the presence of divalent pentasaccharides (Ref. 49). This N-terminal-mediated multimerization was believed to be the main form of galectin-3 oligomerization. Further supporting evidence to this model came from studies using full-length and N-terminally truncated forms of galectin-3. Fermino et al. showed that full-length galectin-3, but not the truncated form Gal-3C, exhibited non-saturable binding to neutrophils and promoted neutrophil activation (Ref. 51). Studies by Halimi et al. confirmed the existence of N-terminal-mediated oligomers, potentially pentamers, induced by the binding of lacto-N-neoTetraose (LNnT) to the CRD. These associations were observed to occur only with the full-length galectin-3. These authors concluded that LNnT binding abolishes the inter-domain interactions between the N- and C-terminal domain of galectin-3 and, consequently, leads to N-type oligomerization (Ref. 48). Apart from the N-type association, another self-association mechanism in the absence of the saccharide ligand, whereby galectin-3 can form multimers via C-terminal (Figure 2D), was demonstrated by Yang et al. (Ref. 52). This C-terminal-dependent multimerization was later confirmed by Lepur et al. who showed that multivalent ligands, such as asialofetuin, can serve as a nucleating agent and induce galectin-3 oligomerization via the CRD (Ref. 53).

Together, these researches indicate that galectin-3 can self-associate through interactions between the N-terminal domains, the C-terminal domains or a combination of both depending on the protein concentration and the availability and type of the binding ligands (Ref. 54).

### Intramolecular interactions between N- and C-terminal domains

In addition to intermolecular associations, there is also evidence showing the existence of interactions between the disordered N-terminal with the C-terminal domains within one galectin-3 molecule. Molecular modelling and NMR studies on hamster galectin-3, which has the same number of collagen-like repeats as human galectin-3, suggested that the galectin-3 N-terminal domain can adopt two folding arrangements. In the first form, it can turn towards the first and last β strands (β1 and β11). In the second configuration, the N-terminal domain was predicted to be situated across the carbohydrate-binding site that contributes to glycan binding (Refs 32, 43). Ippel and colleagues further reported that the N-terminal segment of the galectin-3 interacts transiently with the F-face of CRD and that the residues 23–45 within the N-terminal domain constitute the primary binding epitope (Ref. 55). These interactions between the N-terminal residues and part of the CRD that is not involved in carbohydrate recognition were confirmed in subsequent studies using a range of methods (Ref. 56). This interaction was shown to be primarily driven by both hydrophobic and cation–π forces, suggesting regulatory mechanism for protein self-association and phase separation (Refs 55, 57).

Regarding the relationship between the quaternary structure and the carbohydrate-binding activity, galectin-3 multimerizes on binding by multivalent ligands and that two domains, N-terminal and CRD, contribute to the multimerization and positive binding cooperativity. Moreover, the binding was characterized as bi-phasic in which a rapid binding with lower affinity is followed by a slow phase of higher affinity binding event (Refs 32, 52, 58).

**Figure 3.**
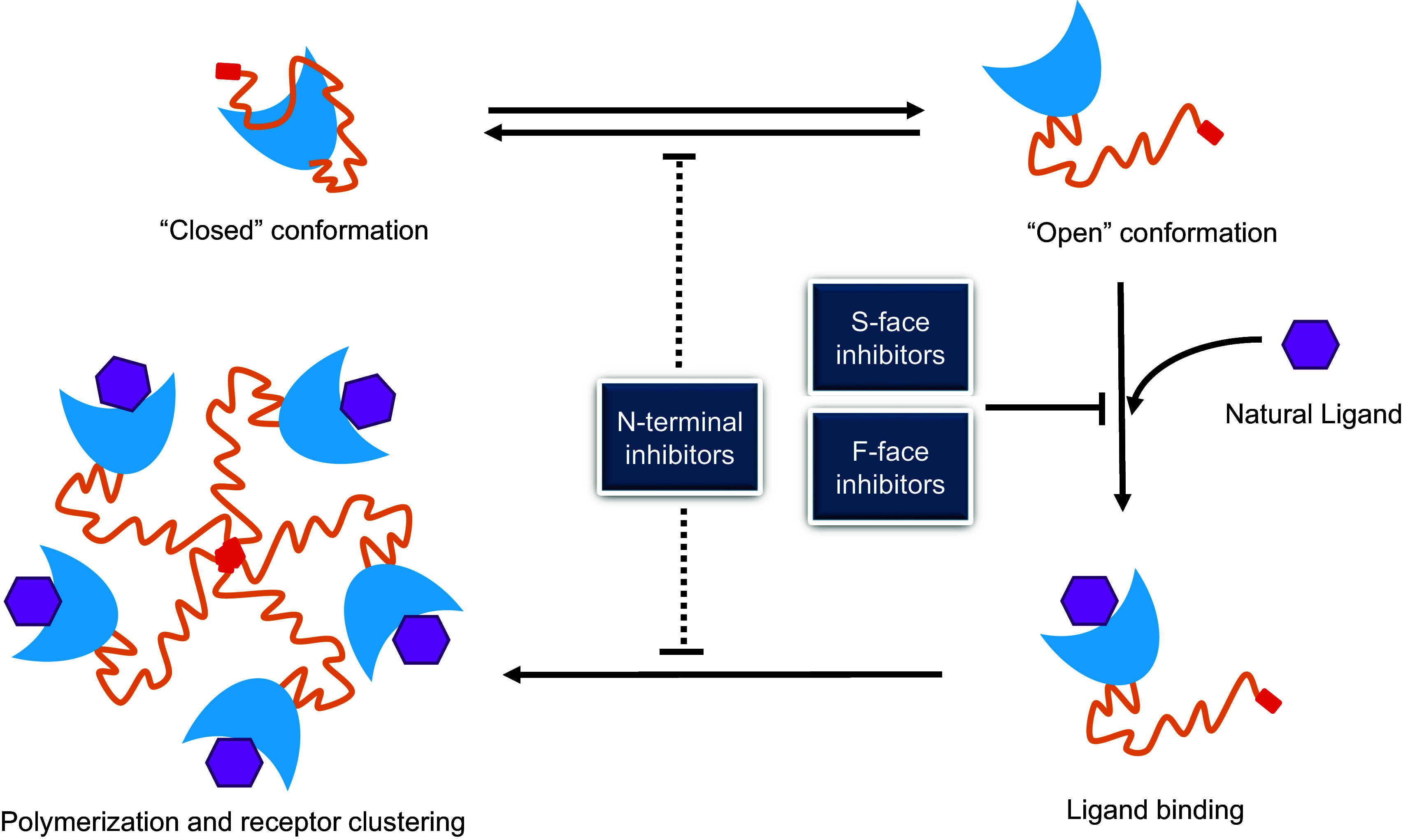
Modes of actions of galectin-3 and galectin-3 antagonists. *Note*: Galectin-3 presents ‘open’ and ‘closed’ conformations in pathophysiological conditions. Galectin-3 binding to its natural ligands on the cell surface, via S-face of CRD, induces galectin-3 polymerization mediated through its N-terminal domain, leading to receptor cell surface clustering and signalling. Galectin-3 inhibitors/antagonists developed against the S- and F-faces CRD prevent galectin-3 binding to its natural ligands directly (S-face inhibitors) or indirectly by changing galectin-3 CRD conformation (F-face inhibitors), while antagonists against the N-terminal domain can inhibit galectin-3 polymerization, receptor clustering and signalling and potentially can also affect galectin-3 ‘open/closed’ conformations. Figure 3. long description.

A bio-activation model with open and closed conformations of galectin-3 was proposed by Suthahar et al. (Ref. 54). This bio-activation model predicts that galectin-3 exists in two states, an inert ‘closed’ conformation held by intramolecular interactions between the N-terminal domain and the F-face of the CRD, and an active ‘open’ conformation formed by intermolecular associations (Ref. 54) (Figure 3). This model suggests that release of the N-terminal domain from the CRD interface is associated with galectin-3 multimerization that promote a spectrum of galectin-3-mediated biological functions.

### Targeting the S-face of the canonical carbohydrate-binding sites of galectin-3 CRD

Given the functional importance of the canonical carbohydrate-binding site, the commonly used strategies in developing galectin-3 antagonists, have naturally been targeting the conserved canonical carbohydrate-binding site, the S-face of the galectin-3 CRD. Pharmacological inhibition of galectin-3 almost exclusively targeted this particular site. Vast majority of the galectin-3 antagonists against this canonical carbohydrate binding site were designed using glycomimetics. These carbohydrate-based blockers can be grouped broadly into two classes based on their molecular size and multivalency, the small molecule synthetic inhibitors and naturally derived, complex and multivalent polysaccharides.

#### Carbohydrate-based small molecule galectin-3 inhibitors

Galectin-3 binds very weakly to galactose, the minimal carbohydrate-binding moiety for galectin-3, with dissociation constant in millimolar range. Extension of galactose structure with other sugar moieties can lead to a significant boost of the galectin-3 binding affinity. For example, the galactose-containing disaccharide Galβ1,4Glc (lactose) binds to galectin-3 (Kd, ~200 μM) nearly 10-fold stronger than galactose (Ref. 59). Further modifications of the glucose molecule in lactose, such as addition of an N-acetyl group as LacNAc type II (Galβ1–4GlcNAc), further enhances its binding affinity (Kd, 30–55 μM) to galectin-3 (Refs 60, 61, 62). The main reason for the weak interaction of galectin-3 with monosaccharide/shorter saccharides is the fact that the galectin-3 carbohydrate-binding interface is formed by shallow indentations on the S-side of the CRD (Ref. 63). The weak interaction of galectin-3 with saccharides is also due to inherent properties of the carbohydrate ligands, which are typically uncharged and possess only limited hydrophobic surfaces that do not promote strong binding affinity (Ref. 60). The primary determinants of selectivity and affinity are hydrogen bonds, van der Waals attractions and hydrophobic interactions between the non-polar regions of the sugar moiety and aromatic amino acid side chains within the binding site (Ref. 63). Improved selectivity and affinity are achieved though extension of the binding site, forming additional contacts as well as water-mediated contacts between galectin-3 and its glycans. A summary on galectin-3 binding regions, affinities, nature of structures and development stages of the galectin-3 antagonists are shown in Table 1.

**Table 1.** Summary of galectin-3 inhibitors/antagonists in pre-clinical and clinical development Table 1. long description.

Inhibitor names	Binding affinities	Nature of inhibitors	Binding sites and specificity	Phase of development	References
3-O-coumarylmethyl- substituted thiodigalactosides derivative 5	91 nM	Thiodigalactoside (di-β-d-galactopyranosyl sulphane) derivatives	CRD (S-face)/strong affinity for Gal–3; binds Gal–1, Gal–2, Gal–7, Gal–8 N, and Gal–9 in low micromolar range and Gal–4C at 210 μM.	Preclinical (reduces fibrotic progression in bleomycin-induced lung fibrosis).	(Ref. 73)
TD139 /GB0139	68 nM	Thiodigalactoside (di-β-d-galactopyranosyl sulphane) derivatives	CRD (S-face)/binds both Gal–3 and Gal–1 in nanomolar range.	Phase Ib/IIa (well tolerated in COVID–19 pneumonitis while decreasing circulating Gal–3 concentration). Phase IIb (IPF) failed primary endpoint.	(Refs 72, 76, 82, 124, 130, 131, 132, 133)
GB1107	37 nM	Galactose-compound conjugates	CRD (S-face)/high affinity for Gal–3 and Gal4C; binds Gal–4 N, Gal–8C, Gal–1 and Gal–9 at 2.4–11 μM and Gal–8 N at 83 μM.	Pre-clinical (efficacious in mouse models of lung adenocarcinoma and fibrosis. Clinical development aborted because of inhibition of hERG potassium channel in heart).	(Refs 74, 75, 82, 134)
GB1211 (Selvigaltin)	25 nM	Galactose-compound conjugates	CRD (S-face)/high affinity for Gal–3 and Gal–4C; binds Gal–4 N, Gal–8C, Gal–1 and Gal–9 at 1.6–8.9 μM and Gal–8 N at 126 μM.	Phase Ib/IIa (reduces liver fibrosis; also positive response when combined with atezolizumab in NSCLC).	(Refs 76, 82, 135, 136)
methyl–2-O-acetyl–3-O-toluoyl-b-d-talopyranoside	550 μM	Talose-compound conjugates	CRD (S-face)/also binds Gal4C with ~threefold lower affinity	Pre-clinical (tool/chemical probe)	(Refs 77, 130)
Methyl-deoxy–2-O-toluoyl–3-N-toluoyl-b-d-talopyranoside	910 μM	Talose-compound conjugates	CRD (S-face)/selective for Gal–3, binds Gal–1 with ~2.5-fold lower affinity.	Pre-clinical (tool/chemical probe)	(Refs 77, 130)
Methyl 2-*O*-(2-nitrobenzoyl)–3-*O*-(4-methylbenzoyl)-β-d-talopyranoside (Cpd018)	8 μM	Talose-compound conjugates	CRD (S-face)/selective for Gal–3, bind Gal–4C with low micromolar affinity and Gal–1 with >200-fold lower affinity.	Pre-clinical (cytotoxic to BCP-ALL cells).	(Refs 77, 130)
lactulosyl-l-leucine	N/A	Glycopeptide	CRD (S-face)/selective Gal–3 and Gal–1	Pre-clinical (reduces lungs and bone metastasis of breast and prostate cancer cells; inhibits cell aggregation and endothelial cell morphogenesis).	(Refs 78, 79, 137)
TFD100	97 pM	Glycopeptide	CRD (S-face)/selective for Gal–3, ~5 times lower affinity for Gal–4 and Gal–9	Pre-clinical (effective against metastatic prostate cancer and suppresses T-cell apoptosis).	(Ref. 91)
GM101/102	Picomolar range (pM)	Recombinant glycopeptide	CRD (S-face)/tested on Gal–3	Pre-clinical (effective against NASH fibrosis and type 2 diabetes)	(Ref. 22)
K2 and L2	18.1 μM (K2) 30.2 μM (L2)	Non-carbohydrate compounds	CRD (S-face)/tested on Gal–3	Pre-clinical (inhibits cancer cell adhesion; anti-angiogenesis and anti-inflammatory properties; reduce tumour growth in chick embryo and murine models; reduce lung metastasis of colon cancer in mice).	(Ref. 81)
MG–257	0.15–0.29 μM	Non-carbohydrate compounds	CRD (S-face)/tested on Gal–3 and Gal–1	Pre-clinical (inhibits Gal–3/TREM2 interactions, reduces amyloid-beta fibrils induced-microglial activation. Impedes neuroinflammation).	(Ref. 80)
Berbamine hydrochloride	N/A	Non-carbohydrate compounds	CRD (F-face)/tested on Gal–3	Pre-clinical (improve motor functions in mouse model of Huntington’s disease).	(Ref. 84)
G3-C12	72 nM	synthetic ~15-mer peptide	CRD (S-face)/tested on Gal–3	Pre-clinical (directs drug to mitochondria of cancer cells).	(Ref. 95)
Citrus segment membrane RG-I pectin	0.32 μM	Polysaccharides	CRD (S-face)/pan-galectin binding.	Pre-clinical (anti-proliferative in human breast and lung cancer cells).	(Refs 108, 110)
Heparin derivatives E and F	1 ~ 1.32 mM	Polysaccharides	CRD (S-face)/tested on Gal–3.	Pre-clinical (anti-metastatic and anti-angiogenic, reduce lung metastasis in mouse models).	(Refs 92, 93)
Heparan sulphate (HS)	Micromolar range	Polysaccharides	CRD (S-face)/tested on Gal–3.	Pre-clinical (inhibit Gal–3 mediated erythrocyte hemagglutination).	(Refs 94, 95)
Davanant	2.9 μM	Polysaccharides	CRD (primarily F-face)/also binds Gal–1.	Phase I/II (mixed results in colorectal cancer; no longer in active development).	(Refs 116, 138)
RG-I–4 (from ginseng pectin)	22.2 nM	Polysaccharides	CRD (S-, F-face and N-terminal)/also binds Gal–1 and Gal–8	Pre-clinical (induces T-cell apoptosis, inhibits cancer cell aggregation and adhesion).	(Refs 86, 112, 117)
GCS–100	N/A	Polysaccharides	Unknown	Phase II (positive signal in phase IIa, but failed to confirm efficacy in phase IIb in chronic kidney disease)	(Refs 120, 139)
Belapectin	2.8 μM	Polysaccharides	CRD (S-face and F-face)/also bind Gal–1.	Phases Ib and IIb/III (positive signal in metastatic melanoma and head and neck cancer when used in combination with pembrolizumab).	(Refs 115, 117, 140)
Hylach (lactose functionalized hyaluronic acid)	N/A	Polysaccharides	CRD (S-face)/docking predicts binding to Gal–3.	Pre-clinical (reduces pro-inflammatory cytokine secretion in human chondrocytes model of osteoarthritis; inhibits fibroblast activation and oxidative stress in COPD and lung fibrosis models).	(Ref. 141)
DP9	N/A	Polysaccharides	CRD (S-face)/docking predicts binding to Gal–3.	Pre-clinical (inhibits pancreatic cancer cell proliferation, angiogenesis and metastasis).	(Ref. 142)
Other MCPs	N/A	Polysaccharides	CRD (S-face and F-face)/tested on Gal–3.	Pre-clinical (inhibit Gal–3-induced red cell hemagglutination and cancer cell aggregation).	(Ref. 109)

**Figure 4.**
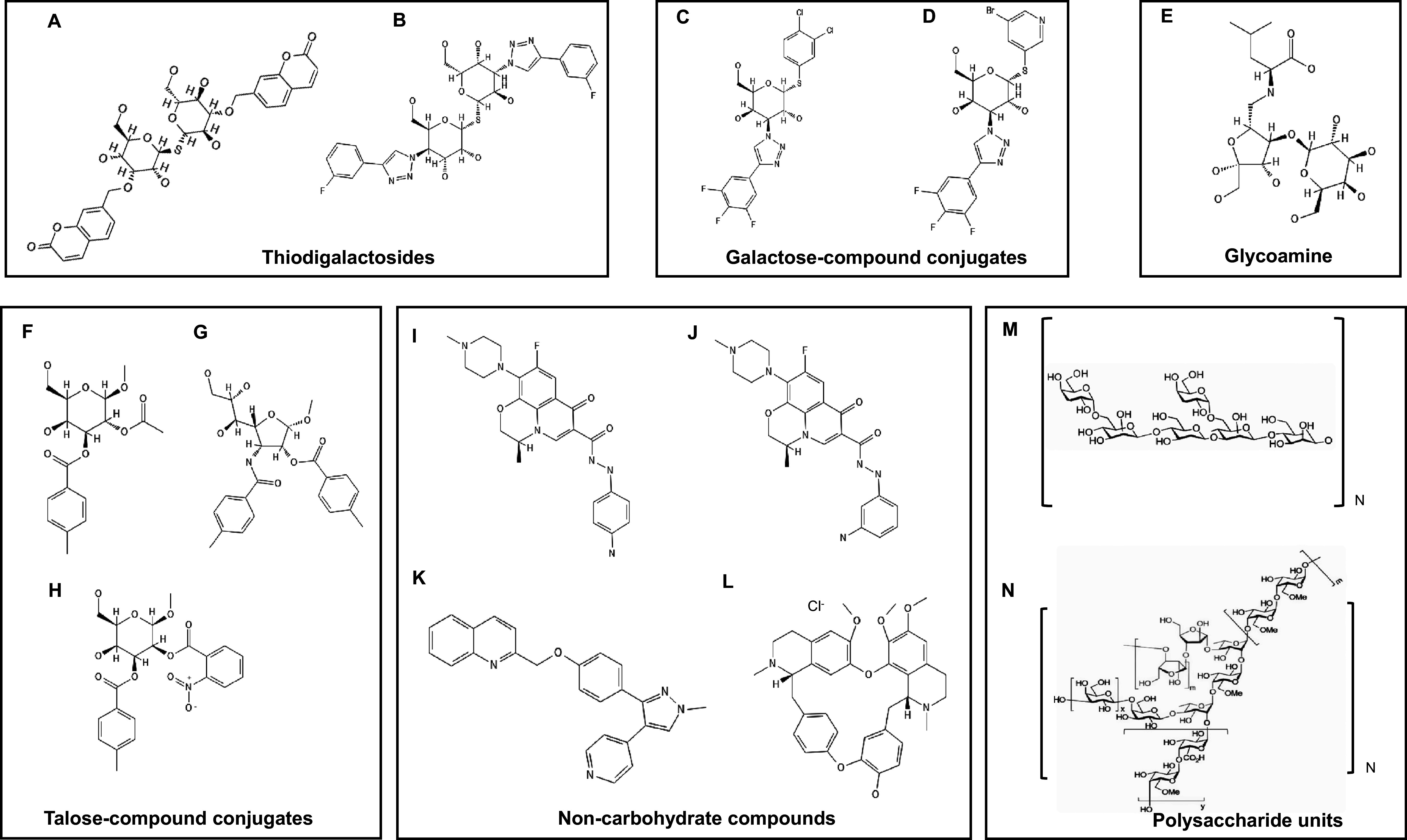
Structures of the galectin-3 inhibitors. *Note*: **A.** *Bis*-3,3′-[(2H-1-benzopyran-2-on-7-yl)methyl]-1,1′-sulphanediyl-di-β-d-galactopyranoside; **B.** 1,1′-sulfanediyl-*bis*-3-deoxy-3-[4-(3-fluorophenyl)-1H-1,2,3-triazol-1-yl]-β-d-galactopyranoside (TD139); **C.** 3,4-dichlorophenyl 3-deoxy-3-[4-(3,4,5-trifluorophenyl)-1H-1,2,3-triazol-1-yl]-1-thio-β-d-galactopyranoside (GB1107); **D.** 5-bromopyridin-3-yl 3-deoxy-1-thio-3-[4-(3,4,5-trifluorophenyl)-1H-1,2,3-triazol-1-yl]-β-d-galactopyranoside (GB1211); **E.** lactulosyl-l-leucine; **F.** methyl 2-O-acetyl-3-O-toluoyl-β-d-talopyranoside; **G.** methyl 3-deoxy-2-O-toluoyl-3-N-toluoyl-β-d-talopyranoside; **H.** methyl 2-O-(2-nitrobenzoyl)-3-O-(4-methylbenzoyl)-β-d-talopyranoside (Cpd018); **I.** K2; **J.** L2; **K.** MG-257; **L.** berbamine hydrochloride; **M.** galactomannan repeating units [(1 → 6)-α-d-galacto-(1 → 4)-β-d-mannan] (red: d-galactopyranoside; black: d-mannopyranoside); **N.** rhamnogalacturonan I (RG-I) (red: d-arabinofuranoside; blue: l-rhamnopyranoside; black: d-galactopyranoside or d-galacturonic acid). Figure 4. long description.

One of the early strategies in the development of galectin-3-targeting antagonists relied on the design of the natural mimicking compounds/inhibitors. Such an approach involved synthesis of small molecule and monovalent ligands based on galactose, lactose or LacNAc scaffold to enable the new compounds not only make more contacts with C–D binding interface on galectin-3, but also interact with residues beyond the C and D subsites to engage interactions with subsites A and B as well as E (Ref. 64). Using such approaches, researchers in Sweden synthesized a library of galactoside-compound chimera inhibitors that were shown to interact with galectin-3 at nM affinity (Ref. 65). These chimeric inhibitors included a series of modified LacNAc derivatives, aromatic lactose-2-O-esters, O-galactosyl aldoximes and their triazole-substituted derivatives as well as mannosyl triazoles (Refs 65, 66, 67, 68, 69). The highly potent LacNAc-derived galectin-3 inhibitors: 3′-amido LacNAc derivative, 3′-triazolyl LacNAc derivative and 3’-O-benyl LacNAc derivative showed to bind galectin-3 with affinity of 320 nM, 660 nM and 13 μM respectively (Ref. 65). These three compounds were designed to extend their interaction into subsites B and A of the carbohydrate binding site on galectin-3. High-affinity bindings were accomplished predominantly by introduction of aromatic moiety to the galactose structure, which formed π–cation interactions with the arginine residue (Arg144) of galectin-3. This arginine residue displays inherent flexibility and adopt various conformations, thus, contribute to greater affinity and specificity (Ref. 60). The arginine–arene attractions emerged as an important factor in enhancing galectin-3 binding affinity (Ref. 70). However, it was the generation of thiodigalactoside (di-β-d-galactopyranosyl sulphane) derivatives (Figure 4), which were designed to interact with all five subsites from A to E, that led to great success. Thiodigalactosides have since been exploited as a promising lead scaffold for the design of small molecule glycomimetics against galectin-3 (Ref. 71). Two most potent galectin-3 ligands, 3,3′-diamido thiodigalactoside and 3,3′-ditriazolyl thiodigalactoside, were demonstrated to bind galectin-3 with K_D_ of 29 nM and 50 nM. The high-affinity interaction of these inhibitors was attributed to symmetrical 3,3′ substitution with aromatic structures that mediated double arginine (Arg144 and Arg186) ligand stacking interactions formed on both ends of the compound in subsites A–B and E on galectin-3 CRD. These compounds were reported to also interact with galectin-9, though the binding affinity was 36-fold weaker (Refs 64, 65). A fluorine-containing analogue of 3,3′-ditriazolyl thiodigalactoside (3,3′-deoxy-3,3′-bis-(4-[m-fluorophenyl]-1H-1,2,3-triazol-1-yl)-thio-digalactoside) named TD139 was shown to bind to galectin-3 with nanomolar affinity. This high binding affinity (Kd, 68 nM) of TD139 with galectin-3 was attributed, like in the parental compound, to extra π–cation interactions with the two arginine residues, Arg144 and Arg186 (Ref. 72).

In 2016, several doubly 3-O-coumarylmethyl-substituted thiodigalactosides were reported to bind to galectin-3 with high affinity and specificity. The most potent one, which bound galectin-3 with affinity of 9 nM, displayed efficacy in a bleomycin-induced mouse model of lung fibrosis. Unfortunately, the compound exhibited poor solubility and required DMSO as co-solvent (Ref. 73).

Further attempts to enhance the binding affinity and selectivity for galectin-3 resulted in the generation of another series of galactose derivatives which bound galectin-3 with low-nanomolar affinity. This enhanced binding affinity was achieved through the introduction of un-natural structural modifications that provided additional contacts with galectin-3 such as fluorine–amide, phenyl–arginine and sulphur–π interactions as well as halogen bonding (Ref. 74). These chimeric compounds (Figure 4) represent another generation of high-affinity galactose-derived galectin-3 inhibitors. The most effective compound GB1107 (3,4-dichlorophenyl 3-deoxy-3-[4(3,4,5-trifluorophenyl)-1H-1,2,3-triazol-1-yl]-1-thio-α-D-galactopyranoside) bound galectin-3 with affinity of 37 nM (Refs 74, 75). GB1107 is characterized by low clearance and good uptake when administered orally in animals. Mice treated with the compound showed inhibition of lung adenocarcinoma growth and metastasis as well as augmented response to a PD-L1 immune checkpoint inhibitor (Refs 75, 76).

All those galectin-3 inhibitors discussed above were aimed for these antagonists to form associations with A–E subsites on galectin-3 CRD. Another interesting approach in designing galectin-3 inhibitors exploited the existence of subsites perpendicular to the natural carbohydrate-binding interface of galectin-3. 2-Sulpho-3-benzamido galactosides were derived from this approach and showed to interact with galectin-3 with dissociation constant of 87 μM (Ref. 65). Molecular modelling suggests that this sulphate group of compounds were engaged in polar interactions with the guanidinium group of Arg144 (Ref. 66). Despite being strong galectin-3 binding, the polar and ionic nature of the sulphate substituents likely limits their in vivo use therapeutically (Ref. 64).

The main disadvantage of these carbohydrate-based inhibitors is their low specificity for galectin-3. Many carbohydrate-based inhibitors that are designed to target galectin-3 often interact with other galectins such as galectin-1 and -9 (Ref. 69). This lack of specificity forced exploration of the unique structural features within galectin-3 molecule to improve binding specificity. The uncovering of a small cavity in galectin-3 enclosed by Arg144 on one side and the S4–S5 loop on the other side has allowed synthesis of highly specific galectin-3 ligands based on the taloside (the C2 epimer of galactose) scaffold such as 2-O-acetyl-3-O-toluoyl-b-d-talopyranoside and -deoxy-2-O-toluoyl-3-N-toluoyl-b-d-talopyranoside. This cavity of galectin-3 is less pronounced in galectin-1. In addition, longer S4–S5 loop and the presence of a histidine residue (His52) within the loop cause the intrusion of the cavity and can hinder the docking of the molecule. It has been shown that addition of hydrophilic substitutions at position O2 of the taloside could further improve specificity for galectin-3 over galectin-1 (Ref. 77).

Other drawbacks of carbohydrate-based small molecule inhibitors are their association with problems of hydrolysis, rapid absorption and metabolism, as well as short half-life (Refs 54, 64). Nevertheless, all the examples discussed above illustrated the possibility of achieving high binding affinity and improved selectivity for galectin-3 by chemically modifying the natural galectin-3 binding glycans with non-carbohydrate structural elements such as triazolyl structures.

#### Glycopeptide galectin-3 inhibitors

Galectin-3-TF antigen interaction is known to be an important factor in driving tumour cell metastatic spreading in cancer. Not surprisingly, compounds mimicking this natural galectin-3 binding glycan have been explored. An example here is represented by lactulosyl-l-leucine (Lac-l-Leu) which combines carbohydrate structures with a short peptide. This small molecular weight TF antigen mimic was demonstrated to inhibit PC-3 prostate cancer bone metastasis in animal model (Ref. 78). In addition, the combination of Lac-L-Leu with the chemotherapeutic drug paclitaxel showed to enhance the sensitivity of taxol-induced apoptosis of breast cancer, both *in* *vitro* and *in* *vivo* (Ref. 79).

#### Non-carbohydrate small molecule galectin-3 inhibitors

More recently, a new generation of non-carbohydrate small molecule galectin-3 inhibitors have been reported (Refs 80, 81). Compounds K2 and L2, which have the same molecular composition (MW = 466) with difference of one -NH_2_ group located at para (K2) or meta (L2) position at one of its aromatic rings, have been shown to bind to galectin-3 at low micromolar affinity (Ref. 81). These isoform compounds (Figures 3, 4) were shown to inhibit galectin-3 binding to its ligand and reduce galectin-3-mediated cancer cell adhesion, invasion, angiogenesis and macrophage secretion of pro-inflammatory cytokines. K2 administration caused significant reduction of galectin-3-mediated tumour growth and metastasis in mice (Ref. 81). Importantly, both compounds showed no detectable cytotoxicity and genotoxicity. Dynamic analysis suggests interaction of these compounds with the S-face of the galectin-3 CRD in which the aromatic atoms of both the tricyclic-fluoro quinolone core and carbohydrazide anilino side chain form the main interactions with galectin-3. The Arg144 residue of galectin-3 CRD was shown to be critically involved in the interaction of these compounds with galectin-3. Interestingly, the Arg144 residue was early reported to also be critical in galectin-3 interaction with carbohydrate-based galectin-3 inhibitors.

Another small molecule compound (MW = 392) named MG-257 with a quinoline–pyrazole scaffold was also reported to bind to galectin-3 at low micromolar affinity and inhibit galectin-3 interaction with myeloid cell surface protein TREM2 (Ref. 80). Molecular dynamics predicted interaction of this compound with the canonical S-face of galectin-3 CRD with involvement of Trp184 and Glu184 residues.

It should be mentioned that although those non-carbohydrate small molecule galectin-3 inhibitors are predicted to interact with the concave side of galectin-3 CRD by molecular docking, the exact positions of their interactions on galectin-3 remain to be experimentally determined. Interestingly, the interaction of K2 and L2 with galectin-3 was demonstrated to induce galectin-3 conformation changes (Ref. 81), while the MG257 interaction was shown to stabilize galectin-3 conformation (Ref. 80). This suggests that the binding sites and modes of action of those non-carbohydrate inhibitors are not the same. The prediction by molecule docking of the involvement of different amino acid residues on the carbohydrate recognition site between these inhibitors is in keeping with this possibility. It is currently unknown whether any of these non-carbohydrate small galectin-3 inhibitors also recognize other galectin members. The chimera carbohydrate based small molecule galectin-3 inhibitors such as GB1211 is known to bind to other galectin family members at substantially lower affinity (Ref. 82). It remains to be determined whether lower binding affinities towards other galectin members also occurs to those non-carbohydrate small molecule galectin-3 inhibitors.

Most recently, berbamine hydrochloride, the hydrochloride salt of berbamine, a natural *bis*-benzylisoquinoline alkaloid isolated from plants like *Berberis amurensis* (Ref. 83), has been reported to be a galectin-3 inhibitor (Ref. 84). Berbamine hydrochloride showed to inhibit galectin-3 self-association through its N-terminal, reduce galectin-3 binding to a microglial surface glycoprotein receptor (TREM2) *in* *vitro* and delay impairment of motor function and progression of neurological deficits in a Huntington’s disease mouse model (Ref. 84). Molecular docking predicted binding of berbamine hydrochloride to the F-face of the galectin-3 CRD near the β7, β8 and β9 strands that induces the structural changes to impair galectin-3 glycan binding (Ref. 84). The study also suggested a positive role of the galectin-3 N-terminal domain in stabilization of berbamine binding to the galectin-3 F-face. Like the other non-carbohydrate small molecule galectin-3 inhibitors, it remains to be determined how specific berbamine hydrochloride is against galectin-3 compared to other galectins. The discovery of berbamine hydrochloride as a galectin-3 inhibitor and its proposed mode of action highlights the importance of galectin-3 N-terminal domain and supports the potential of therapeutic development of galectin-3 antagonists against its non-carbohydrate binding regions.

Like the other small molecule galectin-3 inhibitors, these non-carbohydrate small molecule inhibitors represent promising compounds which could be further optimized in terms of enhancing binding affinity and specificity for galectin-3. One of the advantages of small molecule inhibitors compared to polysaccharide inhibitors is the easiness in characterization of their interactions with galectin-3, including estimation of binding affinity and identification of the exact binding interface. In addition, small molecule synthetic compounds allow controlled, reproducible and large-scale synthesis which are important in drug development. One big advantage of non-carbohydrate small molecule galectin-3 antagonists over carbohydrate-based ones is their potential of being designed with more desired characteristics such as oral bioavailability and improved pharmacokinetics properties that can help to avoid the limitations associated with carbohydrate-based compounds in drug development.

#### Complex polysaccharide galectin-3 inhibitors

Several large complex polysaccharides have been shown to effectively inhibit galectin-3 activities *in* *vitro* and in animals. Like the small synthetic carbohydrate-based inhibitors discussed above, those polysaccharides were generally designed to interact with the galectin-3 canonical carbohydrate-binding site to modulate its activity through competitive inhibition. Polysaccharide inhibitors can improve the relatively weak interactions of galectin-3 with simple carbohydrates through multivalent interactions (Ref. 63). This enhancement in binding affinity is attributed to several factors of galectin multivalency (number of available CRDs), oligomeric state of galectin-3 as well as multivalency of the ligand (Ref. 85). Several plant-derived polysaccharides with exposed galactose residues have been shown to be good galectin-3 inhibitors. Two of the most studied examples are the modified citrus pectin (MCP), derived from acidic modification of the natural pectin, and RG-I-4 from ginseng pectin. These complex polysaccharides were initially believed to interact with the canonical carbohydrate binding site, the S-face, of galectin-3 CRD. Later researches, however, suggest their primarily binding sites are actually on the F-face of galectin-3 CRD (Ref. 86). The F-face interaction of these polysaccharides with galectin-3 will be discussed in more details in later section.

Several other multivalent carbohydrate-based molecules such as neoglycoconjugates were also explored as potential galectin-3 binding inhibitors. These compounds were obtained by coupling lactose-containing carbohydrates to a dendrimer based on the 3,5-di-(2-aminoethoxy) benzoic acid branching unit, resulting in glycodendrimers containing 2, 4 and 8 lactose units (Ref. 87). These glycodendrimers, though showing preferential binding to galectin-3, also bound to galectin-1 (Ref. 87). Small lactose-functionalized poly(amidoamine) (PAMAM) dendrimers were also shown to attenuate aggregation of cancer cells mediated by galectin-3 interactions with TF-bearing MUC1 (Ref. 88). A set of 22 different neo-glycoproteins composed of N-acetyllactosamine or N-diacetyllactosaine-terminated tetrasaccharides conjugated to lysine groups of bovine serum albumin via squaric acid diethyl ester link was shown high-affinity binding to galectin-3 (Ref. 89). It is not yet known whether those compounds are effective on inhibiting galectin-3-mediated actions in vivo.

Some Antarctic fish antifreeze glycoproteins are known to carry multiple numbers of the TF disaccharide (Ref. 90). One TF disaccharide-rich glycopeptide from Pacific cod, designated as TFD_100_, was reported to bind galectin-3 at picomolar affinity. This naturally-derived glycopeptide showed to effectively reduce PC3 prostate cancer adhesion, inhibit galectin-3-mediated angiogenesis and T-cell apoptosis *in* *vitro* and reduce lung metastasis of PC3 cells in nude mice (Ref. 91). These galectin-3 inhibitors have so far not yet reached clinical investigation. This is largely due to the challenges faced by the poor drug-like properties associated with such large, highly glycosylated, heterogeneous glycoproteins/glycopeptides as well as the manufacturing challenges to produce consistent batches. Such large multivalent glycoproteins/glycopeptides also pose off-target immunological risk. This highlights an important principle in galectin-3 biology that affinity alone is not sufficient for drug development.

Several chemically modified (desulphated) heparin derivatives were also reported to be good galectin-3 inhibitors (Ref. 92). These chemically modified heparin derivatives were shown to inhibit galectin-3 binding to TF-bearing glycans and decrease galectin-3-mediated cancer cell adhesion *in* *vitro* and reduce lung metastasis in mice grafted with human melanoma and colon cancer cells. Interestingly, despite lacking galactose moiety, these modified heparin derivatives were shown to bind to the canonical carbohydrate binding site on the S-face of CRD (Ref. 92), albeit the binding was weak in low mM range (Ref. 93). Several glycosaminoglycans, such as chondroitin sulphate, have also been reported to bind to galectin-3 at the conserved carbohydrate-binding site on the S-face with nanomolar affinity (Ref. 94).

#### Peptide-based galectin-3 inhibitors

Two synthetic peptides named G3-A9 and G3-C12 with amino acid sequences PQNSKIPGPTFLDPH and ANTPCGPYTHDCPVKR, respectively, were reported to bind galectin-3 with affinity of 17–80 nM (Ref. 95). These peptides were shown to block galectin-3 interactions with TF, inhibit rolling and adhesion of breast carcinoma cells to the endothelium *in* *vitro* and reduce lung metastasis in mice (Refs 95, 96, 97).

Two peptides derived from the galectin-3 CRD sequence, which consist of the key amino acid residues responsible for galectin-3 glycan binding, were also explored as competitive galectin-3 binding inhibitors. One of these peptides is composed of 10 amino acids 152–162 GNDVAFHFNPR, whereas the other harbours seven amino acids 177–183 LDNNWGR of galectin-3 CRD. Both peptides showed inhibition of galectin-3 binding to neoglycoprotein (Lac-BSA), with the longer peptide showing more potent than the shorter one (Ref. 98). The concentrations of the peptides used in this assay were however quite high (0.5 and 2.0 mM) which likely restricts their potential of therapeutic applications.

As a competitive inhibitor, the truncated C-terminal galectin-3 (Gal3C) has been demonstrated to promote tumour cell anoikis, decrease cancer cell adhesions *in* *vitro* (Ref. 99) and reduce tumour growth and metastasis of breast cancer cells in mice (Ref. 100). Administration of Gal-3C with bortezomib showed to suppress 94% tumour growth in a xenograft mouse model of multiple myeloma (Ref. 101).

### Targeting the F-face of the galectin-3 CRD

The non-canonical carbohydrate binding site on the F-face CRD is increasingly reported to be involved in galectin-3 interaction with ligands and critical in galectin-3-mediated cellular activities. CD146 is a natural galectin-3 binding ligands on the surface of endothelial cells (Ref. 102) and melanoma cells (Ref. 103). Binding of galectin-3 to CD146 on endothelial cells promotes endothelial secretion of pro-metastatic cytokines that increase tumour cell adhesion and angiogenesis (Ref. 102). Interaction of galectin-3 with CD146 on melanoma cells induced CD146 dimerization and cell migration and invasion (Ref. 103). NMR analysis revealed the involvement of the CRD F-face in interaction with CD146 (Ref. 104). It is likely that binding to the F-face of galectin-3 CRD changes galectin-3 conformation leading to decreased galectin-3 interaction with its natural ligands on its canonical carbohydrate-binding site on S-face.

MCP, generated through controlled pH and thermal treatment of citrus pectin, is among the most extensively studied multivalent galectin-3 antagonists. Citrus pectin is a large and complex polysaccharide composed of galactose, arabinose and anhydrogalacturonic acid (Ref. 105). There are two major types of pectins: galacturonans and rhamnogalacturonans (RG-1). Galacturonans are composed of linear (1,4)-linked α-d-GalA residues and can be substituted with saccharides such as xylose, fucose and apiose. Rhamnogalacturonan has a backbone of repeating disaccharide unit [−4-α-d-GalA-1,2-α-l-Rha-1-], often with substitutions at C4 of the Rha moiety with chains built up by β-d-galactose, α-l-arabinose or both (Ref. 106). These pectin polysaccharides were shown to inhibit cancer cell aggregation, invasion and angiogenesis, enhance tumour cell sensitivity to chemotherapy in vitro (Refs 97, 105, 107, 108) and reduce tumour growth and metastasis in mice (Refs 109, 110).

Detailed biochemical analysis of galectin-3 interactions with citrus pectin and synthetic oligosaccharide fragments, derived from the side chains as well as backbone of pectins, showed surprisingly very poor inhibitory activity against galectin-3. An early research suggested that pectin-derived polysaccharides do not interact with the canonical carbohydrate-binding site of galectin-3 (Ref. 106). This was supported by the observation that the vast majority of galactose residues within citrus pectin-derived polysaccharides exist in Galβ1–4Gal configuration which is unfavourable for galectin-3 binding. NMR analysis has revealed interaction of these large polysaccharides primarily with the F-face rather than the S-face of galectin-3 CRD, though some of its carbohydrate epitopes can also bind to the S-face (Ref. 111).

Rhamnogalacturonan I (RG-I)-rich fragment from ginseng pectin (RG-1-4) is another pectin-derived polysaccharide inhibitor. RG-I binds to galectin-3 with an affinity of 22.2 nM and can reduce galectin-3-induced hemagglutination (Ref. 112), block cancer cell adhesion and aggregation. RG-I was reported to interact with a non-canonical carbohydrate-binding site on galectin-3 (Ref. 106). Structure–activity relationship analysis demonstrated the requirement of the galactan side chains in RG-I action, whereas the arabinan moieties were suggested to regulate RG-I activity, either positively or negatively depending on their localization within the pectin molecule (Ref. 112).

Two α-galactomannan polysaccharide derivatives, GM-CT-01 (Davanat) from guar seeds and GR-MD-02 (Belapectin) from apples, have been studied extensively as galectin-3 antagonists. These polysaccharides bind to galectin-3 with low micromolar affinity (2.9 and 2.8 μM respectively) (Ref. 96). Phase II clinical trial of Davanat demonstrated improved survival of colorectal cancer patient survival. Belapectin also showed good efficacy in attenuation of inflammation, hepatocyte injury and fibrosis in animal models (Ref. 113) and was well tolerated in human (Ref. 114). In a Phase IIa clinical trial in patients with NASH, cirrhosis and portal hypertension, bi-weekly infusions of belapectin for 52 weeks reduced HVPG and development of varices in a sub-group of patients without oesophageal varices, though no significant effect on reduction in HVPG or fibrosis was observed compared with placebo (Ref. 115). These demonstrations of modest and inconsistent efficacy and the shift of standard of care to combination chemotherapy by Davanat and the heterogeneous and insufficient efficacy in the overall population by belapectin have prevented these polysaccharide inhibitors to progress further in clinical development (Ref. 115).

The structure of Davanat was estimated to be approximately 59 kDa (Ref. 116). Its large size makes it less susceptible to fast metabolism and clearance from circulation. Davanat binds to galectin-3 as well as to galectin-1 (Ref. 116). Like other pectin polysaccharides, Davanat was shown to interact with galectin-3 and galectin-1 at sites different from the conventional carbohydrate-binding site (Refs 106, 117) and reduce galectin-3-ligand binding on the S-face (Ref. 117).

Collectively, these investigations suggest that the observed effects on galectin-3 of pectin and galactomannan derivatives are not totally due to their interaction with the canonical carbohydrate-binding site of galectin-3 to directly block galectin-3–carbohydrate interactions. They can bind to the F-face CRD and introduce galectin-3 conformational changes and reduction of galectin-3 binding to its natural ligands through the S-face. Structural deviations of galectin-3 conformation can occur within loops in close proximity to the conserved carbohydrate-binding interface and detectable rearrangements of these loops was indeed observed (Ref. 118). This confirms the structural flexibility of galectin-3 CRD and supports a likely structural deviation of galectin-3 in response to polysaccharide antagonists. The report that Davanat binding to galectin-3 introduced a conformational transition within the β-sandwich structure and the interconnecting loops (Ref. 117) is in good support to this possibility. The increased revelation of an influence of F-face binding on galectin-3 recognition to its glycans on its S-face suggests that a combination of galectin-3 antagonists against the S- and F-faces (e.g. an antibody against the residues on the F-face and an inhibitor to the S-face) may potentially offer greater efficacy in therapeutic treatment of galectin-3-mediated pathologies.

A big challenge in developing naturally occurring polysaccharide galectin-3 inhibitors as therapeutic agents is their high polydispersity and heterogeneity (Ref. 12). This challenge significantly limits effective development of these naturally occurring polysaccharides. Consequently, focus was later turned into MCP-derived polysaccharides. One of the MCP-derived polysaccharides, GCS-100, showed to inhibit cancer cell growth and enhance tumour cell sensitivity to immunochemotherapy *in vitro* and in animal models (Refs 12, 97, 119). It demonstrated good safety and activity in phase II clinical trials for the treatment of relapsed chronic lymphocytic leukaemia (CLL) and multiple myeloma (Refs 120, 121). However, in spite of showing good tolerability and no serious adverse effects, further clinical development of GCS-100 was recently discontinued, due to the complex nature of the polysaccharides and the difficulty in their accurate characterization (Ref. 71).

### Targeting the N-terminal domain of galectin-3

The highly flexible region of galectin-3 is known to control galectin-3-multimerization in response to ligand binding. When sprayed onto the surface covered with mica, an N-terminal fragment (residues 1–125) of galectin-3 was shown to self-associate into amyloid-like fibrils (Ref. 43). N-terminal self-association and association with CRD also occurs in solution (Ref. 56). Receptor clustering and signalling induced by galectin-3 N-terminal multimerization is a classical mode of action of galectin-3 in response to ligand binding in many galectin-3-mediated cellular activities in pathological conditions (Refs 10, 57, 103, 122, 123). Given the unique tri-modular structure and its mode of actions in diseases, galectin-3 N-terminal potentially offers another targeting region for therapeutic development of galectin-3 antagonists. Although antagonists against this galectin-3 segment may not directly affect galectin-3-receptor interactions, they can prevent galectin-3-mediated receptor clustering and signalling, thus inhibiting galectin-3-mediated cellular activities in diseases. One of the advantages of targeting the N-terminal domain of galectin-3 is its selectivity and specificity. Galectin-3 is the only member in the galectin family that possesses this flexible N-terminal feature. Antagonists that are designed to target this unique segment of galectin-3 would be expected to avoid cross-reaction with other galectin members thus offering greater specificity.

### Lessons from galectin-3 antagonist clinical trials

Despite encouraging results from early-phase clinical trials, no galectin-3 inhibitor has yet progressed to phase III so far. This lack of progression, however, does not reflect a lack of biological relevance, rather a combination of target biology complexity, drug design limitations and clinical trial challenges.

The galectin-3 inhibitors undergone clinical trials so far are all carbohydrate-based compounds [polysaccharide, e.g., belapectin (GR-MD-02) (Ref. 115), or carbohydrate-compound conjugates, e.g., TD139/GB0139, GB1211) (Refs 124, 125)]. Such carbohydrate-based inhibitors often suffer from the typical limitations associated with carbohydrates as drugs of poor physico-chemical properties (e.g. poor bioavailability, tissue penetration and biodistribution). They are biologically interesting but often pharmacologically sub-optimal.

Galectin-3 functions both inside and outside cells. These carbohydrate-based inhibitors all target the galectibn-3 CRD and mostly work on extracellular galectin-3. There is no evidence that any of these galectin-3 inhibitors can gain effective intracellular exposure. Large polysaccharide inhibitors (e.g. MCP, belapectin) are highly hydrophilic and could not enter cells, while the small molecule galectin-3 inhibitors so far tested in clinical trials are also highly polarized compounds (e.g. TD139, GB1211). Such molecules generally have poor passive membrane permeability. Their use therefore can lead to only partial inhibition of the galectin-3 actions. Moreover, galectin-3 is not a single dominant driver but part of a network of fibrosis/inflammation pathways and inhibiting it alone may produce modest or context-dependent effects. Some trial designs also raised issues of inadequate indication selection (e.g. late-stage fibrosis where reversal is difficult, monotherapy where combination therapy is likely required or lack of inadequate patient stratification where high inter-patient variability can prevail). Lessons learned from the early clinical trials have enabled the development of galectin-3 antagonists from complex polysaccharides (first generation) to carbohydrate-compound conjugates (second generation) to non-carbohydrate small molecule compounds (third generation) and the strategic rethinking of the design of clinical trials. It can be envisaged that good galectin-3 antagonists are expected to demonstrate true target engagement in tissues, be used in the right biological context (e.g. early-stage disease, stratified galectin-3 high patients) and possibly in combination. The present clinical trials of GB1211 in combination with the checkpoint inhibitor anti-PD-L1 in non-small cell lung cancer and head and neck cancer are strategic refinements. The outcome of these ongoing trials will help to shape the development strategy further and aid the progress of galectin-3 antagonist development.

## Concluding remarks and future perspectives

As a multi-mode regulator in cancer, inflammation and fibrosis-associated diseases, galectin-3 is a well-recognized therapeutic target in multiple disease areas. Several approaches have been explored in developing galectin-3-targeted therapeutic inhibitors/antagonists, and several have shown encouraging results in early phases clinical trials. All the current approaches are targeting the canonical carbohydrate-binding site on the S-face of galectin-3 CRD. Increasing evidence has indicated that the non-carbohydrate-binding site on the F-face CRD and the N-terminal segment can significantly influence the galectin-3 activities, by either reducing galectin-3 binding to its ligands on the S-face or by preventing galectin-3-mediated receptor clustering and signalling. This suggests that in addition to the canonical carbohydrate-binding interface on the S-face, the F-face of galectin-3 CRD and the flexible N-terminal region are also targetable in developing galectin-3-targeted therapeutics (Figure 3). Given the high degree of structural similarities of the CRDs between members of galectins, galectin-3 antagonists developed against the N-terminal domain of galectin-3 could avoid cross-reactivity with other galectin members and offer greater target specificity. Moreover, galectin-3 antagonists that could interact with more than one segment of galectin-3, or a combination of antagonists against different segments of galectin-3, may also provide improved efficacy in treatment of galectin-3-mediated pathologies.

It is known that proteolytic cleavage of galectin-3 can occur in its N-terminal segment, specifically within the Pro-Gly-Tyr repeat (collagen-like) linker region that connects the N-terminal oligomerization domain to the C-terminal carbohydrate recognition domain (CRD) (Refs 39, 126). The most common cleavage site by matrix metalloproteinases (especially MMP-2 and MMP-9) occurs at Ala62-Tyr63, leading to a −22 kDa C-terminal (CRD intact) and a ~9 kDa N-terminal fragments of galectin-3. Galectin-3 cleavage contributes to tumour growth, angiogenesis and apoptosis resistance (Ref. 127). Although it is not yet known how galectin-3 exerts these effects at the molecular level extracellularly, it is conceivable that galectin-3 cleavage could expose otherwise conceded intra- and inter-molecule binding sites to allow galectin-3 interaction with additional ligands. Considering the existence of different galectin-3 conformations, cleavage of galectin-3 at Ala62-Tyr63 would remove its N-terminal domain to enable galectin-3 to be in an ‘open’ conformation for interaction with its naturally binding ligands. Therapeutically, S- and F-faces targeted galectin-3 antagonists would be expected to work effectively against such galectin-3-mediated activities, while N-terminal-targeted antagonists would not.

The anti-apoptotic activity of galectin-3 is a well-recognized intracellular galectin-3 action in cancer (Ref. 128). This anti-apoptotic galectin-3 action was shown to be mostly independent on galectin-3 carbohydrate- recognition through protein–protein interactions (Ref. 129). It is not yet known how its non-carbohydrate-binding regions contribute to such heterotypic protein–protein interaction in apoptosis regulation, more studies in this area are needed. These discoveries, however, stress the validity of therapeutic development against different regions of galectin-3 and the need for consideration of context-dependent applications of galectin-3 antagonists.

Targeting galectin-3 is attractive therapeutically because it sits at the intersection of inflammation, fibrosis, immunity and cancer. This makes it a single-mode intervention that can impact multiple pathological pathways simultaneously – especially useful in complex diseases. The same pleiotropy of galectin-3 actions, however, also poses challenges for galectin-3-targeted drug development. The actions of galectin-3 are often overlapped with other galectin members. Inhibition of galectin-3 actions can be partially compensated by other galectins and could also lead to opposing biological functions (e.g. pro- and anti-inflammatory functions) depending on cell type, localization (intracellular vs. extracellular) and timing and context of diseases (e.g. acute vs. chronic inflammation). The mixed clinical outcomes and limited efficacy so far observed in clinical trials are a manifesto of such challenges. This context dependency is a major translational barrier in therapeutic development of galectin-3 antagonists. A strategy of patient stratification and use of combination therapy likely can help to improve the efficacy of galectin-3-targeted therapies.

Overall, galectin-3 represents an attractive therapeutic target across a broad spectrum of diseases. Although the development of galectin-3-targeted therapies remains at an early stage and continues to face challenges, ongoing advances in our understanding of its mechanisms of actions at cellular, molecular and structural levels – together with emerging technologies – are expected to drive the design and development of more selective and effective galectin-3 antagonists.

## Abbreviations

CRD: carbohydrate recognition domain
Gal-3C: C-terminal domain of galectin-3
Lac: lactose
LacNAc: N-acetyl-d-lactosamine
TF antigen: Thomsen–Friedenreich antigen

## Long descriptions

### Figure 1. Long description

Panel A shows a molecular surface representation of the galectin-3 C R D. The top concave S-face is light pink and contains a blue stick-model lactose molecule. The bottom convex F-face is teal.

Panel B shows a green ribbon cartoon of the same protein structure.

* The S-face consists of six beta-strands labeled in red as S 1 through S 6, corresponding to yellow labels beta 1, beta 10, beta 3, beta 4, beta 5, and beta 6.

* The F-face consists of five beta-strands labeled in dark blue as F 1 through F 5, corresponding to yellow labels beta 11, beta 2, beta 7, beta 8, and beta 9.

* Carbohydrate-binding subsites are labeled A, B, C, D, and E from bottom to top along the S-face.

* The blue lactose molecule is positioned at subsites C and D.

* The N-terminus and C-terminus are labeled at the bottom of the ribbon structure.

Navigate back to Figure 1..

### Figure 2. Long description

Panel A shows a schematic monomer. From bottom to top, it consists of a red rectangular short N-terminal stretch of approximately 21 a a, an orange zigzag representing 9 collagen-like repeats of approximately 91 a a, and a blue crescent-shaped Carbohydrate Recognition Domain C R D of approximately 138 a a. Brackets group the bottom two sections as the N-terminal domain and the top section as the C-terminal domain.

Panel B displays a ribbon diagram of the full-length protein. A red cartoon structure is overlaid with a grey crystal structure of the C R D. A blue stick representation of a lactose molecule is bound at the top. A 180-degree rotation arrow separates two views of the model.

Panel C illustrates N-type multimerization. At the center, five orange zigzag N-terminal domains are joined at a red star-like hub. Each orange tail extends outward to a blue C R D. Two large green circles representing ligands are shown, with grey ovals attached to them by black lines, which are being bound by the blue C R D units.

Panel D illustrates C-type self-association. A linear chain of blue C R D units is formed, with their orange and red N-terminal tails extending outward. A large green ligand with attached grey ovals is shown interacting with the side of the C R D chain.

Navigate back to Figure 2..

### Figure 3. Long description

The diagram consists of four main states of galectin-3 and a central inhibitory section.

* Top-Left: A single galectin-3 molecule in a Closed conformation, where the orange N-terminal tail is folded against the blue C R D crescent.

* Top-Right: An Open conformation where the N-terminal tail is extended away from the C R D. Horizontal arrows indicate a reversible transition between the closed and open states.

* Bottom-Right: Ligand binding state. A purple hexagonal Natural Ligand is shown binding to the concave S-face of the blue C R D. A vertical arrow from the Open conformation leads here, while a T-bar indicates inhibition of this step.

* Bottom-Left: Polymerization and receptor clustering. Five galectin-3 molecules are arranged in a pentameric circle, joined at the center by their N-terminal domains, with purple ligands bound to each C R D. A horizontal arrow points from the ligand-bound state to this cluster.

* Center: Three blue boxes identify inhibitor types. N-terminal inhibitors are positioned on a vertical dashed line that blocks the transition between closed/open states and the polymerization process. S-face inhibitors and F-face inhibitors are grouped together, with a T-bar pointing to the arrow for ligand binding, indicating they block the interaction between the C R D and the natural ligand.

Navigate back to Figure 3..

### Table 1. Long description

The table contains six columns.

* Row 1: 3-O-coumarylmethyl- substituted thiodigalactosides derivative 5; 91 n M; Thiodigalactoside derivatives; C R D S-face with strong affinity for Gal-3; Preclinical for lung fibrosis.

* Row 2: T D 1 3 9 / G B 0 1 3 9; 68 n M; Thiodigalactoside derivatives; C R D S-face binding Gal-3 and Gal-1; Phase I b / I I a for C O V I D-19 pneumonitis.

* Row 3: G B 1 1 0 7; 37 n M; Galactose-compound conjugates; C R D S-face high affinity for Gal-3 and Gal 4 C; Pre-clinical for lung adenocarcinoma.

* Row 4: G B 1 2 1 1 Selvigaltin; 25 n M; Galactose-compound conjugates; C R D S-face high affinity for Gal-3 and Gal-4 C; Phase I b / I I a for liver fibrosis.

* Row 5: methyl-2-O-acetyl-3-O-toluoyl-b-D-talopyranoside; 550 micro M; Talose-compound conjugates; C R D S-face; Pre-clinical tool.

* Row 6: Methyl-deoxy-2-O-toluoyl-3-N-toluoyl-b-D-talopyranoside; 910 micro M; Talose-compound conjugates; C R D S-face selective for Gal-3; Pre-clinical tool.

* Row 7: C p d 0 1 8; 8 micro M; Talose-compound conjugates; C R D S-face selective for Gal-3; Pre-clinical for B C P-A L L cells.

* Row 8: lactulosyl-L-leucine; N / A; Glycopeptide; C R D S-face selective Gal-3 and Gal-1; Pre-clinical for breast and prostate cancer metastasis.

* Row 9: T F D 1 0 0; 97 p M; Glycopeptide; C R D S-face selective for Gal-3; Pre-clinical for metastatic prostate cancer.

* Row 10: G M 1 0 1 / 1 0 2; Picomolar range; Recombinant glycopeptide; C R D S-face; Pre-clinical for N A S H fibrosis.

* Row 11: K 2 and L 2; 18.1 micro M and 30.2 micro M; Non-carbohydrate compounds; C R D S-face; Pre-clinical for cancer cell adhesion.

* Row 12: M G-2 5 7; 0.15-0.29 micro M; Non-carbohydrate compounds; C R D S-face; Pre-clinical for neuroinflammation.

* Row 13: Berbamine hydrochloride; N / A; Non-carbohydrate compounds; C R D F-face; Pre-clinical for Huntington's disease.

* Row 14: G 3-C 1 2; 72 n M; synthetic 15-mer peptide; C R D S-face; Pre-clinical for drug delivery to mitochondria.

* Row 15: Citrus segment membrane R G-I pectin; 0.32 micro M; Polysaccharides; C R D S-face pan-galectin binding; Pre-clinical anti-proliferative.

* Row 16: Heparin derivatives E and F; 1 to 1.32 m M; Polysaccharides; C R D S-face; Pre-clinical anti-metastatic.

* Row 17: Heparan sulphate H S; Micromolar range; Polysaccharides; C R D S-face; Pre-clinical for erythrocyte hemagglutination.

* Row 18: Davanant; 2.9 micro M; Polysaccharides; C R D primarily F-face; Phase I / I I for colorectal cancer.

* Row 19: R G-I-4; 22.2 n M; Polysaccharides; C R D S-, F-face and N-terminal; Pre-clinical for T-cell apoptosis.

* Row 20: G C S-1 0 0; N / A; Polysaccharides; Unknown; Phase I I for chronic kidney disease.

* Row 21: Belapectin; 2.8 micro M; Polysaccharides; C R D S-face and F-face; Phases I b and I I b / I I I for metastatic melanoma.

* Row 22: Hylach; N / A; Polysaccharides; C R D S-face; Pre-clinical for osteoarthritis.

* Row 23: D P 9; N / A; Polysaccharides; C R D S-face; Pre-clinical for pancreatic cancer.

* Row 24: Other M C Ps; N / A; Polysaccharides; C R D S-face and F-face; Pre-clinical for red cell hemagglutination.

Navigate back to Table 1..

### Figure 4. Long description

The diagram is organized into six distinct boxes.

* Top-left box (Thiodigalactosides): Contains structures A and B. Structure A is a symmetrical molecule with two galactopyranoside rings linked by a sulfur atom, each substituted with a benzopyran-2-one group. Structure B (T D 139) features two galactopyranoside rings with fluorophenyl-triazolyl substitutions.

* Top-middle box (Galactose-compound conjugates): Contains structures C (G B 1107) and D (G B 1211). Both feature a single galactopyranoside ring with a triazolyl-trifluorophenyl group at the 3-position and a thio-linked dichlorophenyl (C) or bromopyridinyl (D) group at the 1-position.

* Top-right box (Glycoamine): Contains structure E, lactulosyl-L-leucine, showing a disaccharide linked to a leucine amino acid chain.

* Bottom-left box (Talose-compound conjugates): Contains structures F, G, and H (Cpd 018). These are based on talopyranoside rings with various acetyl, toluoyl, and nitrobenzoyl ester or amide substitutions.

* Bottom-middle box (Non-carbohydrate compounds): Contains structures I (K 2), J (L 2), K (M G-257), and L (berbamine hydrochloride). These are complex polycyclic aromatic and heterocyclic molecules including quinolines and alkaloids.

* Bottom-right box (Polysaccharide units): Contains structures M and N. Structure M shows repeating units of galactomannan with alpha-1,6 and beta-1,4 linkages. Structure N shows the complex branched structure of rhamnogalacturonan I (R G-I) involving rhamnose, galactose, and arabinose units.

Navigate back to Figure 4..
